# Safety of Red Blood Cell Transfusion Using Small Central Lines in Neonates: An *in vitro* Non-inferiority Study

**DOI:** 10.3389/fped.2021.606611

**Published:** 2021-03-03

**Authors:** Flavia Rosa-Mangeret, Sophie Waldvogel-Abramowski, Riccardo E. Pfister, Olivier Baud, Sébastien Fau

**Affiliations:** ^1^Division of Neonatology, Geneva University Hospital (HUG), Geneva, Switzerland; ^2^Division of Hematology, Geneva University Hospital (HUG), Geneva, Switzerland

**Keywords:** neonatal care, neonatal transfusion, premature (babies), quality of care/care delivery, blood transfusion, transfusion—alternative strategies

## Abstract

**Aim:** This study aimed to investigate the safety of transfusing red blood cell concentrates (RBCCs) through small [24 gauge (24G)] and extra-small [28 gauge [28G)] peripherally inserted central catheters (PICCs), according to guidelines of transfusion practice in Switzerland.

**Methods:** We performed a non-inferiority *in vitro* study to assess the safety of transfusing RBCC for 4 h at a 4 ml/h speed through 24G silicone and 28G polyurethane PICC lines, compared with a peripheral 24G short catheter. The primary endpoint was hemolysis percentage. Secondary endpoints were catheter occlusion, inline pressure, and potassium and lactate values.

**Results:** For the primary outcome, hemolysis values were not statistically different among catheter groups (0.06% variation, *p* = 0.95) or over time (2.75% variation, *p* = 0.72). The highest hemolysis values in both 24G and 28G PICCs were below the non-inferiority predefined margin. We did not observe catheter occlusion. Inline pressure varied between catheters but followed the same pattern of rapid increase followed by stabilization. Potassium and lactate measurements were not statistically different among tested catheters (0.139% variation, *p* = 0.98 for potassium and 0.062%, *p* = 0.96 for lactates).

**Conclusions:** This study shows that RBCC transfusion performed *in vitro* through 24G silicone and 28G polyurethane PICC lines is feasible without detectable hemolysis or pressure concerns. Also, it adds that, concerning hemolysis, transfusion of RBCC in small and extra-small PICC lines is non-inferior to peripheral short 24G catheters. Clinical prospective assessment in preterm infants is needed to confirm these data further.

## Introduction

Peripherally inserted central catheters (PICCs), usually ranging from 24 gauge (G) to 28G, are routinely used in very preterm infants for parenteral nutrition and drug infusion. They can be inserted at the bedside and maintained for several weeks. Their small diameter is also suitable for the most immature neonates, and the central positioning allows infusing high osmolality solutions (1).

Very preterm infants are at high risk of anemia due to impaired erythropoietin production, repeated blood draws, reduced red blood cell life span, iron depletion, and rapid growth. As a result, 80% of very low birth weight (VLBW) and 80–95% of extremely low birth weight (ELBW) neonates need at least one blood transfusion before discharge (2, 3). Potential risks of catheter occlusion and hemolysis exist when considering red blood cell concentrate (RBCC) transfusion through 24/28G PICC lines (1). However, securing a peripheral IV line for transfusions can be challenging and can lead to clinical instability in a critically ill neonate, moving some physicians toward performing them through an available PICC. To the best of our knowledge, there are no clear guidelines on transfusions using neonatal PICC lines, while there is also no evidence that using these devices with this purpose would be hazardous.

Few studies analyzed the feasibility and safety of transfusing RBCC through extra-small (27G) (4, 5) and small (24G) PICCs (6). However, no study compared the safety of transfusing RBCC using the polyurethane and silicone smallest PICC lines with the regularly used 24G short catheters, in conditions simulating a preterm neonate transfusion in the neonatal intensive care unit (NICU).

To understand this issue's relevance in Switzerland, we invited the nine Swiss level III NICU to answer a small self-administered questionnaire regarding PICC usage (available in the Supplementary Material). It revealed that 4/9 (44.4%) had already performed blood transfusion through 28G PICC lines. Among them, only one center declared that catheter blockage occurred. Furthermore, 7/9 (77.8%) units stated they would be willing to use catheters for transfusion if further safety evidence exists.

We then performed a non-inferiority *in vitro* study comparing the smallest PICCs available in Switzerland with the standard peripheral 24G short catheter. We hypothesize that transfusing RBCC through either small (24G) or extra-small (28G) PICC lines is as safe as transfusing RBCC through peripheral 24G short catheters in the NICU environment. The primary objective was to provide evidence for RBCC transfusion safety through extra-small PICC lines considering hemolysis, catheter blockage, and inline pressure levels.

## Materials and Methods

### Setting

We performed a non-inferiority *in vitro* study to assess the safety of transfusing RBCC through small PICC lines compared with a peripheral 24G short catheter. We performed this study at Geneva University Hospital NICU. The Geneva Cantonal Ethics Commission waived the study (BASEC 2018−00823).

### Red Blood Cell Concentrate Transfusion Setup

**Figure 1:**
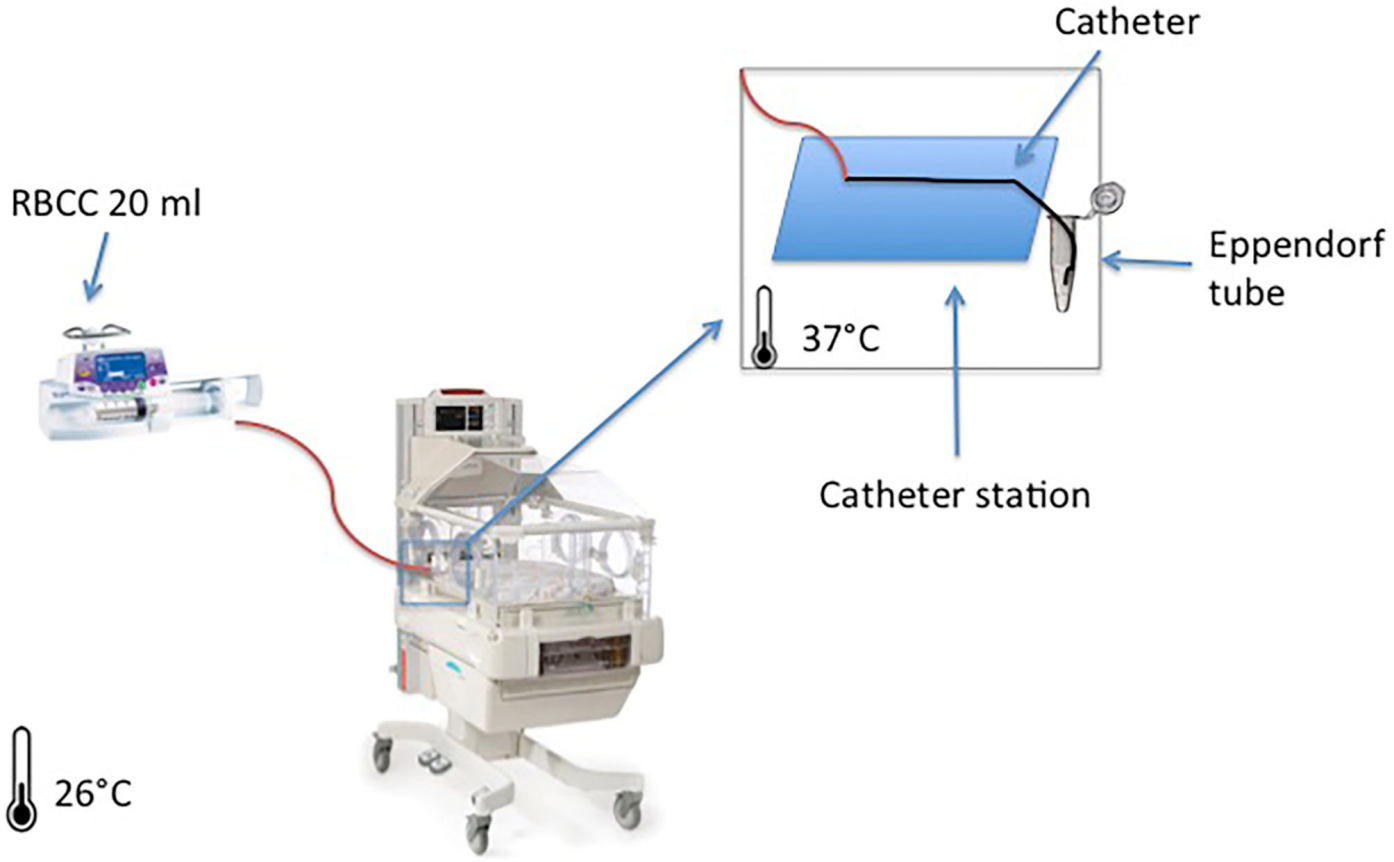
System setup overview: the figure represents one red blood cell concentrate (RBCC) infusion through a peripherally inserted central catheter (PICC) line that lies on a support catheter station with the tip inserted on a collector tube filled in with NaCl 0.9% for sampling. The same setup was used for the three groups at the same time.

To do a mock neonatal RBCC transfusion, we performed the blood transfusion as per our unit protocol, into an incubator with the air temperature set at 37°C, considering the average body temperature of the neonate to be 37°C (Figure 1). In the setting, the incubator simulated the neonate, and the three different catheters underwent simultaneous standard RBCC transfusion in the incubator:

- peripheral 24G short catheter (24G BD Insyte-W™, Utah, USA), used as the control group (CTR);
- polyurethane catheter Premicath 1 Fr/28G 20 cm (Vygon Schweiz GmbH, Aachen, Germany), used as intervention Group #1 (PICC28);
- silicone catheter Epicutaneo 2 2 Fr/24G 30 cm (Vygon Schweiz GmbH, Aachen, Germany), used as intervention Group #2 (PICC24).

For each catheter, we added to the standard transfusion setup a three-way stopcock on the catheter entrance connected to a syringe pump with an embedded inline pressure sensor Alaris CC^®^ (Becton Dickinson, Eysins, Switzerland). A data logger software registered inline pressure every 2 s. A minimal flow of 0.1 ml/h of NaCl 0.9% ran through this pressure measurement line.

The filtered RBCCs were gamma-irradiated immediately (25 Gray) before the procedure, and they were not older than 14 days. We calculated the volume and infusion rate based on a neonate of 1,000 g, who would need 15 ml/kg of RBCC transfusion infused within 4 h.

### Procedure

Upon arrival, RBCC rested at room temperature (26°C) for at least 30 min, and then sampling for hemoglobin, hematocrit, potassium, and free hemoglobin took place before splitting it into three aliquots of 20 ml in 50 ml syringes.

Meanwhile, the three groups received a continuous infusion of parenteral nutrition (per 100 ml: amino-acid 3 g; glucose: 10.8 g; Na: 2 mmol; K: 1 mmol; Ca: 1.1 mmol; PO_4_: 0.86 mmol; osmolarity: 1,000 mOsm/L) for 1 h, at a 4 ml/h speed, followed by a manual flush of 1 ml of NaCl 0.9%.

After parenteral nutrition infusion, RBCC infusion started simultaneously in all groups, at 4 ml/h for 4 h on the syringe pumps (Module DPS Fresenius Vial, Brézins, France). In order to avoid clotting, we placed catheter tips into an isotonic solution (NaCl 0.9%), avoiding direct contact between the red cells and air; and by the end of each hour (H1, H2, H3, and H4), we placed catheters tips into an Eppendorf Tube^®^ 3810X 1.5 ml vial (Eppendorf AG, 22331 Hamburg, Germany) filled with 0.5 ml of NaCl 0.9% to complete 1.5 ml of total sample volume (Figure 1).

After that, we immediately analyzed samples for hemoglobin, hematocrit, and potassium (ABL835 Flex Radiometer Medical Inc., Brønshøj, Denmark) and centrifuged the remaining, in room temperature, at 2,000 rpm for 10 min; we collected 400 μl of the supernatant and froze it at −20°C until free hemoglobin testing (Spectrophotometer Biochrom Libra S70, Holliston, USA). Hemolysis in the RBCC bag was assessed (hemoglobin, hematocrit, and free hemoglobin) before and immediately after the procedure.

At the end of transfusion, all the lines were flushed with 1 ml of NaCl 0.9%, and the parenteral nutrition was restarted in the three systems for at least 1 h to determine pressure variation and possible catheter occlusion.

### Outcomes

The primary outcome was hemolysis, measured as indicated in the European Guidelines (7): % hemolysis = [free hemoglobin (g/L) × (100 – hematocrit (%)]/total hemoglobin.

The secondary outcomes were as follows:

- potassium and lactate serum concentrations at baseline, H1, H2, H3, and H4;
- complete catheter occlusion during and after infusion, defined as inline pressure superior to 600 mmHg with a flush NaCl 0.9% in the 4 ml/h speed;
- inline pressure for each catheter during and after transfusion;

While lactate is not a hemolysis marker, it is considered a surrogate marker for blood storage (8). Hence, we assumed it was relevant to report its variation over time in our study.

### Statistics

For sample size calculation, we considered a limit of the hemolysis parameter below 0.8% (9), and a non-inferiority margin of 0.2%. According to published data, the mean hemolysis for RBCC bags after 2 weeks of storage is 0.23 ± 0.12% (9). The reached sample size was eight separate RBCC transfusions, considering that the mean hemolysis at the end of the transfusion is at least equal to the control and intervention groups, reaching a significance level of 2.5% and 90% power. We compared the hemolysis values at the end of RBCC transfusion (H4) between the control and intervention groups using a one-way ANOVA and Friedman test for paired measures. We also compared hemolysis among catheters and over time, using repeated-measures two-way ANOVA with Geisser–Greenhouse correction. Hemolysis in RBCC bags was compared before and after the procedure using a Wilcoxon matched-pairs signed-rank test.

Secondary outcomes include catheter occlusion, inline pressure comparison before, during (at the steady state), and after RBCC infusion. Potassium and lactate hourly measures were analyzed using repeated-measures two-way ANOVA with Geisser–Greenhouse correction. Statistical analyses used GraphPad PRISM version 6.0 (San Diego, CA).

## Results

### Primary Outcome

**Figure 2:**
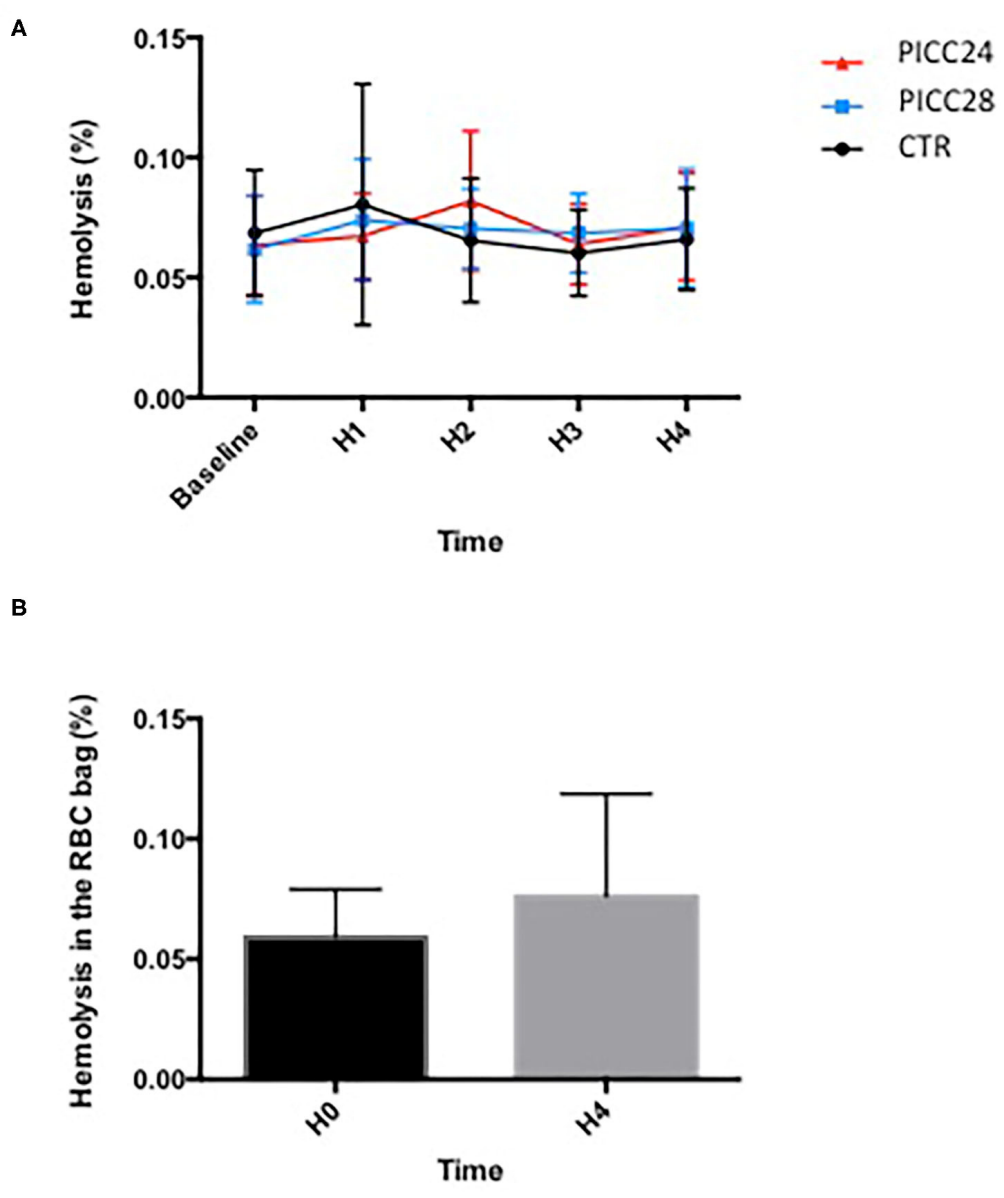
Comparison of hemolysis over time during a 4 h transfusion according to IV line used. **(A)** Mean hemolysis for each catheter assessed each hour; data (mean ± SEM) were compared using a two-way ANOVA with time and catheter type as variables. **(B)** Comparison of hemolysis values (%) in red blood cell concentrate (RBCC) bag between baseline (H0) and the end of transfusion (H4). Data (mean ± SD) were compared using a Wilcoxon matched-pairs signed-rank test.

Difference in means [standard deviations (SD)] of hemolysis values measured at the end of the transfusion procedures (H4) were not statistically significant between the control group [0.0675% (0.0205)], PICC28 [0.0700% (0.0239)], and PICC24 [0.0700% (0.02390)] (*p* = 0.99). For both intervention catheters (PICC28 and PICC24), the higher-margin values were significantly lower than the predefined non-inferiority margin of 0.2%. Figure 2A shows the mean hemolysis values in the three groups from the baseline to the end of transfusion. Comparing the hemolysis values between groups through the whole procedure on repeated-measures two-way ANOVA further confirms that hemolysis values were not statistically changed among catheter groups (0.06% variation, *p* = 0.95) or over time (2.75% variation, *p* = 0.72). The mean hemolysis in the RBCC that remained in the RBCC before and after transfusion was not found statistically different (0.0595% vs. 0.0706%, *p* = 0.11) (Figure 2B). The detailed data are available in the Supplementary Materials.

### Secondary Outcomes

**Figure 3:**
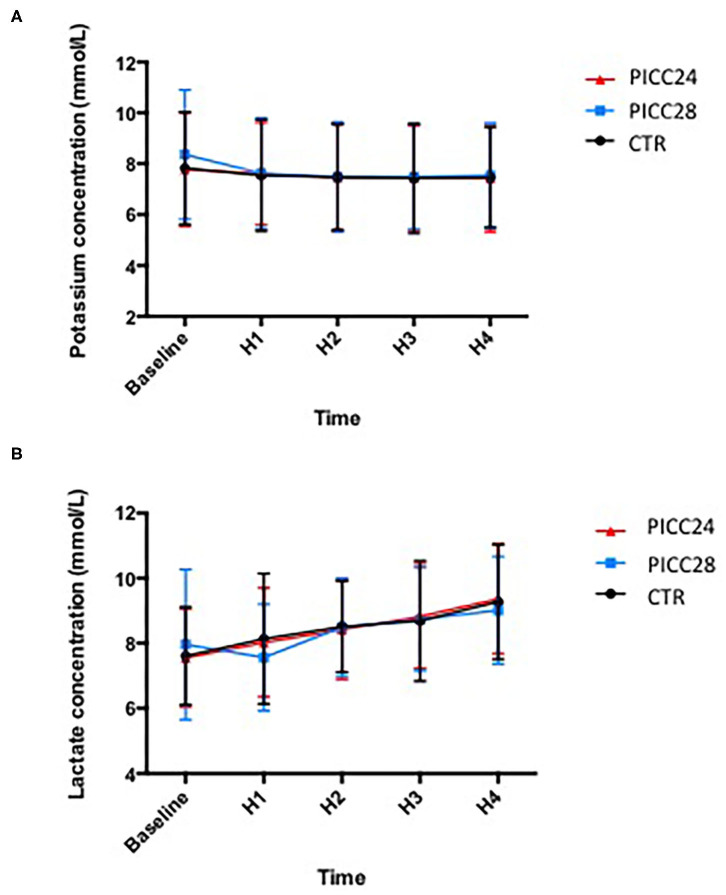
Mean potassium **(A)** and lactate **(B)** concentrations in each IV line group during red blood cell concentrate (RBCC) transfusion.

The potassium and lactate measurements analyzed on a two-way ANOVA revealed a non-significant variation among the three catheter groups (0.139% variation, *p* = 0.98 for potassium and 0.062%, *p* = 0.96 for lactates). Lactate increased significantly in all experimental groups overtime during the transfusion process (10.7% variation, *p* < 0.001). In contrast, potassium measurements remained remarkably stable (Figure 3).

**Figure 4:**
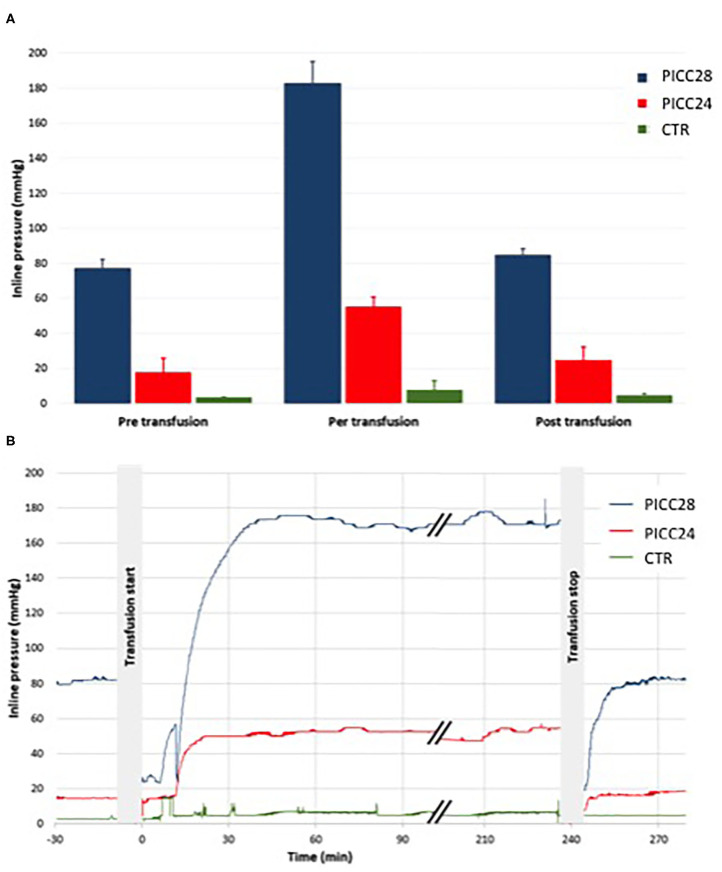
Mean pressure within the lines before, during, and after the transfusion. Comparisons within IV line groups **(A)** and typical traces **(B)** in PICC28 line (blue), PICC24 line (red), and CTR peripheral control line (green).

Catheter occlusion did not happen during RBCC transfusions or parenteral nutrition infusion in the eight separate experiments. The recorded inline pressures were very reproducible among five experiments for the PICC28 group and four experiments for both the PICC24 and control groups (Figure 4A). During RBCC infusion, there was a rapid increase of pressures in the system, likely due to high blood viscosity, being the highest in the PICC28 group with a rise from 77 ± 5 mmHg during parenteral infusion to 183 ± 12 mmHg during transfusion. In the PICC24 group, pressure increased from 18 ± 8 to 55 ± 6 mmHg. In contrast, the pressure increase was limited from 3.3 ± 0.6 to 7.6 ± 5.3 mmHg in the control group (Figure 4B). Infusion of parenteral nutrition at the end of RBCC transfusion allowed, in all cases, a drop in pressure to similar values measured before the RBCC transfusion (84 ± 4, 24 ± 7, and 4.5 ± 1.0 mmHg for PICC28, PICC24, and control lines, respectively).

## Discussion

RBCC transfusions are common interventions in very preterm infants admitted in NICUs and are dependent on available IV lines. In contrast to PICC lines usually already present when a transfusion is needed, peripheral IV lines are not; securing them to an already sick neonate is a challenge. In this *in vitro* non-inferiority study, for the primary outcome of hemolysis, transfusing RBCC in 24 and 28G PICCs was non-inferior to standard 24G short catheter. For the secondary outcomes, we have not detected catheter occlusion; inline pressures were measured within our pre-established limits; and there was no statistically significant difference in potassium and lactate concentrations among experimental groups during RBCC transfusion.

These observations are of interest in light of our Swiss national survey, which exhibits that most NICUs (7/9) would be ready to perform RBCC transfusions using small and extra-small PICC lines if further evidence of feasibility and safety available and some 44.4% (4/9) had already done it. This contrasts with a previous extensive survey performed in the United States, in which out of 186 NICUs, only five used (2.7%) PICC lines to transfuse blood products (10).

Our data show that free hemoglobin concentrations were found similar in PICC lines compared with peripheral IV lines. Also, no detectable adverse events were recorded. In a previous report, Repa et al. have tested *in vitro* the safety of RBCC transfusions in 27G 20 and 30 cm polyurethane PICC lines, without significant hemolysis (5). In another retrospective study, transfusion through a 27G PICC line was found feasible in 38 neonates, while free hemoglobin was not measured in this study, hemoglobin levels increased, and cardiovascular parameters remained stable without increase in potassium levels (4). Our data are consistent with those of previous studies and further support that transfusing RBCC through a 28G polyurethane 20 cm PICC line or a 24G silicone 30 cm PICC line is at least as safe as transfusing RBCC though a 24G short catheter.

Considering the inline pressure, the RBCC transfusion induced a substantial pressure increase. The blood viscosity infused at 4 ml/h through the PICC28 line generated pressures as high as 180 mmHg, far below 300 mmHg, which we and others consider as the safety threshold (11, 12). The rapid pressure drop after transfusion to the range of pre-RBCC transfusion values suggests that PICC resistance remained unchanged and is consistent with the absence of catheter occlusion, even partial, during the transfusion.

This *in vitro* study has several limitations. First, even if we set a system as close as possible to a neonatal RBCC transfusion with body temperature at 37°C and using same equipment and transfusion procedure as in our patients, it remains an *in vitro* study design and needs to be further completed by a clinical prospective study. Second, the speed of 4 ml/h during transfusion, fitting with standard care in a 1,000 g infant, appears to be the highest to avoid 300 mmHg of inline pressure usually considered a limit for safety reasons. We did not test lower speeds considering that they would not be clinically relevant. Third, we used a parenteral mixture with relatively low osmolarity, and catheters were unused for each experiment, all factors that may reduce the risk of catheter occlusion. Finally, we performed the RBCC transfusion alone and not in combination with simultaneous parenteral nutrition, which would not be recommended due to possible damage of RBC (13). However, most neonates needing transfusions are likely to be fed orally, and the sickest neonates will mostly have a two-lumen IV line.

In conclusion, this study showed that *in vitro* RBCC transfusion performed through 24 to 28G neonatal PICC lines was non-inferior to 24G short catheters, with no further evidence of catheter occlusion. These results might be useful in light of a practice that already exists in several NICUs, but safety of RBCC transfusion through PICC lines remains to be confirmed in a prospective clinical study.

## Data Availability Statement

The original contributions presented in the study are included in the article/Supplementary Material, further inquiries can be directed to the corresponding author/s.

## Author's Note

Red blood cell transfusions are a common intervention in very premature infants. Usually, they require a peripheral intravenous line, which can be very challenging to secure. Although very small neonates routinely have peripherally inserted central catheters (PICCs) to receive intravenous nutrition and medication infusion, only few studies are available regarding the safety of using PICCs for red blood cell transfusions. This study assessed the safety of performing blood transfusions through the smallest PICCs available (24 and 28 gauge) using an *in vitro* model that mimics preterm infants' environment, and its results might improve neonatal care to date.

## Author Contributions

FR-M designed the study, wrote the study protocol, performed all pilot tests and the main tests, and wrote the first draft and the last version. SW-A was the expert consultant, responsible to reach the ideal set up, provided the Red Blood Cell Concentrates, participated in the analysis of the samples and results, and edited the manuscript. RP was part of the study design, protocol, the conception and distribution of the survey, and read and edited manuscript. OB was part of the study design, writing of the protocol, ethics committee submission, survey conception, pilots conception, final setup, the statistics, analysis of results, and manuscript. SF was part of the setup conception, the monitoring up of pressures during transfusions, analysis of results, and manuscript editing. All authors revised the first draft of the manuscript and approved the final version.

## Conflict of Interest

The authors declare that the research was conducted in the absence of any commercial or financial relationships that could be construed as a potential conflict of interest.

## References

[1] Mccay AS Elliott EC Walden M. PICC placement in the neonate. N Engl J Med. (2014) 370:e17. 10.1056/NEJMvcm1101914 24620893

[2] Maier RF Sonntag J Walka MM Liu G Metze BC Obladen M. Changing practices of red blood cell transfusions in infants with birth weights less than 1000g. J Pediatr. (2000) 136:220–4. 10.1016/S0022-3476(00)70105-3 10657829

[3] Widness JA Seward VJ Kromer IJ Burmeister LF Bell EF Strauss RG. Changing patterns of red blood cell transfusion in very low birth weight infants. J Pediatr. (1996) 129:680–7. 10.1016/S0022-3476(96)70150-6 8917234

[4] Repa A Mayerhofer M Worel N Cardona F Deindl P Pollak A. Blood transfusions using 27 gauge PICC lines: a retrospective clinical study on safety and feasibility. Klin Padiatr. (2014) 226:3–7. 10.1055/s-0033-1363244 24435791

[5] Repa A Mayerhofer M Cardona F Worel N Deindl P Pollak A. Safety of blood transfusions using 27 gauge neonatal PICC lines : an *in vitro* study on hemolysis. Klin Padiatr. (2013) 225:379–82. 10.1055/s-0033-1355329 24158888

[6] Wong ECC Schreiber S Criss VR LaFleur B Rais-Bahrami K Short B. Feasibility of red blood cell transfusion through small bore central venous catheters used in neonates. Pediatr Crit Care Med. (2004) 5:69–74. 10.1097/01.PCC.0000102225.49058.4B 14697112

[7] Mayr WR. Guide to the preparation, use and quality assurance of blood components, 13th Edition. Vox Sang. (2017) 93:279. 10.1111/j.1423-0410.2007.00965.x8607148

[8] Council of Europe. Guide to the Preparation, Use and Quality Assurance. (2011). Available online at: http://www.centronazionalesangue.it/sites/default/files/guida_edqm_16_edizione.pdf

[9] Serrano K Chen D Hansen AL Levin E Turner TR Kurach JDR. The effect of timing of gamma-irradiation on hemolysis and potassium release in leukoreduced red cell concentrates stored in SAGM. Vox Sang. (2014) 106:379–81. 10.1111/vox.12112 24330144

[10] Sharpe E Pettit J Ellsbury DL. A national survey of neonatal Peripherally Inserted Central Catheter (PICC) Practices. Adv. Neonatal Care. (2013) 13:55–74. 10.1097/ANC.0b013e318278b907 23360860

[11] Keay S Callander C. The safe use of infusion devices. Contin Educ Anaesth Crit Care Pain. (2004) 4:81–5. 10.1093/bjaceaccp/mkh022

[12] Pisciotto PT Wong ECC. Chapter 11 – Technical considerations/mechanical devices. In: Hillyer CD Strauss RG Luban NLC editors. Handbook of Pediatric Transfusion Medicine. Academic Press (2004). p. 121–30. 10.1016/B978-012348776-6/50014-5

[13] Sowemimo-Coke SO. RBC hemolysis during processing.pdf. Transfus Med Rev. (2002) 16:46–60. 10.1053/tmrv.2002.29404 11788929

