# Supplementary material for: Safety of Red Blood Cell Transfusion Using Small Central Lines in Neonates: An in vitro Non-inferiority Study
Source: Front Pediatr. 2021 Mar 3;9:606611. doi: 10.3389/fped.2021.606611 (PMC7968454; doi:10.3389/fped.2021.606611)
Supplement: Supplementary file 1 [file Table_1.DOCX]

# Blood Transfusion over PICC lines in Switzerland

Survey on current Swiss transfusion practices of Red Blood Cells (RBC) in neonates with PICC lines.

The full questionnaire will take about 2 minutes of your time. It is addressed to paediatricians and neonatologists with a fair experience (>1-2 years) of their unit's practices. It should reflect the units practice and not a personal opinion unless stated.

Thank you for helping us!

Riccardo Pfister & Flavia Mangeret

*Obligatoire

1.

Canton *

2.

Please state level of your unit *

level III

level IIa

level IIb

other

3.

If you wish feed-back, please write your e-mail

4.

In your unit, do you use size 28G/1Fr PICC lines? (If you do not use PICC lines at all, you do not need to fill the rest of the form, answer only this question and submit) *

Very often

Often

Rarely

Never

5.

If possible give brand (s) of PICC lines used in your unit

6.

In your unit, do you perform Red Blood Cells transfusion in neonates using 28G/1Fr PICC lines? (If you do not transfuse over PICC lines at all, you do not need to fill section 2, answer the next two questions and submit) *

Often

Rarely

Never

7.

Would you use 28G/1Fr PICC lines for blood transfusion if considered safe? *

Yes

No

Transfusion over 28G/1Fr PICC line

8.

In your unit, if you perform a RBC transfusion on a 28G/1Fr PICC line, do you remove PICC line after transfusion?

Mostly

Rarely

Never

9.

In your unit, after a RBC transfusion over a 28G/1Fr PICC line, do you make any kind of biological in quality control in the patient (i.e. hemolysis markers)

Yes

No

10.

If you use a quality control after PICC line transfusion, please state which biological marker you use

11.

In your unit, when performing a RBC transfusion over a 28G/1Fr PICC line, how often the catheter gets blocked before ending the transfusion?

Often

Rarely

Never

12.

When performing a RBC transfusion over a 28G/1Fr PICC line, have you experienced any other types of incidents related to the transfusion over the PICC line?

Often

Rarely

Never

13.

Please specify incidents observed following transfusion over a 28G/1Fr PICC line

14.

We would be happy for your comments:

Thank you overmuch for completing the survey! Please submit now
