# Supplementary material for: Safety of Red Blood Cell Transfusion Using Small Central Lines in Neonates: An in vitro Non-inferiority Study
Source: Front Pediatr. 2021 Mar 3;9:606611. doi: 10.3389/fped.2021.606611 (PMC7968454; doi:10.3389/fped.2021.606611)
Supplement: Supplementary file 2 [file Table_2.DOCX]

Survey on Transfusion of Packed red blood cell in 28 G picc lines

**Flavia Rosa-Mangeret^1^; Riccardo Pfister^1^; Olivier Baud^1^**

1. Division of Neonatology, Geneva University Hospital (HUG), CH-1211 Geneva, Switzerland
2. Division of Hematology, Geneva University Hospital (HUG), CH-1211 Geneva, Switzerland

#### INTRODUCTION

In neonatal intensive care units (NICUs), PICC lines are routinely used with many advantages as they can be inserted at the patient's bedside and maintained for several weeks. Their lumen's small diameter is suitable for neonates delivered extremely preterm, and the central positioning allows infusing high osmolality solutions, including parenteral nutrition.

Premature neonates weighing less than 1000g are at high risk of anemia, and red blood cell concentrate (RBCC) transfusion is frequent in this population. Securing a peripheral IV line in a sick neonate can be challenging, leading some NICUs to perform RBCC transfusion through small 24 and 28G PICC lines.

***AIM:***

To assess the Swiss neonatal tertiary NICUs practice regarding blood transfusions using PICC lines.

***MATERIALS AND METHODS:***

We performed a national survey of all 9 tertiary NICUs in Switzerland through a self-administered questionnaire.

This questionnaire consisted of 14 questions concerning: demographic information (3), catheter information (2), and their practice and opinion concerning RBCC transfusions through 28G PICC.

#### RESULTS:

All tertiary NICUs answered the questions, and there was no missing data. Among the 9 NICUs, 4 (44,4%) had already performed blood transfusion through 28G PICC lines. Seven NICUs (77,8%) declared to be willing to use PICC lines for transfusion if further safety evidence exists.

Out of the 4 units that reported performing this practice, all declared removing the line after transfusion and not performing any post-transfusion quality control. None of the 4 NICUs reported incidents related to transfusion. Catheter blockage was reported by one NICU.

#### CONCLUSIONS:

According to this survey, most of the Swiss tertiary units would be interested in performing RBCC transfusion through 28G PICC lines, supporting the relevance of further investigation on RBCC transfusions through 28 G PICC lines in vitro.
