# Supplementary material for: Safety of Red Blood Cell Transfusion Using Small Central Lines in Neonates: An in vitro Non-inferiority Study
Source: Front Pediatr. 2021 Mar 3;9:606611. doi: 10.3389/fped.2021.606611 (PMC7968454; doi:10.3389/fped.2021.606611)
Supplement: Supplementary file 4 [file Table_4.DOCX]

Supplementary Table 4: Comparison of hemolysis over time during a 4-hour RBCC transfusion according to IV lines used. CTR means 24G short catheter. Central lines tested were PICC28 and PICC24.

|  | Baseline | | H1 | | H2 | | H3 | | H4 | |
| --- | --- | --- | --- | --- | --- | --- | --- | --- | --- | --- |
|  | Mean | SD | Mean | SD | Mean | SD | Mean | SD | Mean | SD |
| CTR (N=8) | 0,068 | 0,026 | 0,080 | 0,050 | 0,065 | 0,026 | 0,060 | 0,018 | 0,066 | 0,021 |
| PICC28 (N=8) | 0,062 | 0,022 | 0,074 | 0,025 | 0,070 | 0,016 | 0,068 | 0,016 | 0,070 | 0,025 |
| PICC24 (N=8) | 0,0635 | 0,021 | 0,067 | 0,018 | 0,082 | 0,029 | 0,064 | 0,017 | 0,071 | 0,022 |

| **Two-way RM ANOVA analysis** |  | |
| --- | --- | --- |
| Alpha | 0,05 |  |
|  |  |  |
| Source of Variation | % of total variation | P value |
| Interaction | 3,452 | 0,751 |
| Catheter type | 0,060 | 0,955 |
| Time | 2,750 | 0,717 |

Supplementary Table 5: Comparison of potassium concentrations in each IV lines group during RBCC transfusion.

|  | Baseline | | H1 | | H2 | | H3 | | H4 | |
| --- | --- | --- | --- | --- | --- | --- | --- | --- | --- | --- |
|  | Mean | SD | Mean | SD | Mean | SD | Mean | SD | Mean | SD |
| CTR (N=8) | 7,825 | 2,202 | 7,538 | 2,190 | 7,475 | 2,075 | 7,425 | 2,163 | 7,463 | 1,972 |
| PICC28 (N=8) | 8,363 | 2,538 | 7,625 | 2,188 | 7,488 | 2,166 | 7,488 | 2,068 | 7,550 | 2,057 |
| PICC24 (N=8) | 7,775 | 2,226 | 7,600 | 2,010 | 7,463 | 2,093 | 7,438 | 2,049 | 7,425 | 2,101 |

| **Two-way RM ANOVA analysis** |  | |
| --- | --- | --- |
| Alpha | 0,05 |  |
|  |  |  |
| Source of Variation | % of total variation | P value |
| Interaction | 0,232 | >0,9999 |
| Catheter type | 0,140 | 0,928 |
| Time | 0,996 | 0,900 |

Supplementary Table 6: Comparison of lactate concentrations in each IV lines group during RBCC transfusion.

|  | Baseline | | H1 | | H2 | | H3 | | H4 | |
| --- | --- | --- | --- | --- | --- | --- | --- | --- | --- | --- |
|  | Mean | SD | Mean | SD | Mean | SD | Mean | SD | Mean | SD |
| CTR (N=8) | 7,613 | 1,505 | 8,138 | 2,003 | 8,513 | 1,399 | 8,688 | 1,849 | 9,275 | 1,763 |
| PICC28 (N=8) | 7,963 | 2,311 | 7,563 | 1,637 | 8,488 | 1,515 | 8,738 | 1,586 | 9,013 | 1,654 |
| PICC24 (N=8) | 7,550 | 1,506 | 8,025 | 1,675 | 8,425 | 1,534 | 8,825 | 1,604 | 9,363 | 1,681 |

| **Two-way RM ANOVA analysis** |  | |
| --- | --- | --- |
| Alpha | 0,05 |  |
|  |  |  |
| Source of Variation | % of total variation | P value |
| Interaction | 0,793 | 0,342 |
| Catheter type | 0,062 | 0,992 |
| Time | 10,650 | <0,0001 |

Supplemental Table 4: Comparison of hemolysis values (%) in RBCC bag between baseline (H0) and the end of transfusion (H4). Note than one bag analysis was lost (N=7)

| H0 | H4 |
| --- | --- |
| 0,0839 | 0,0972 |
| 0,0535 | 0,0639 |
| 0,0505 | 0,0495 |
| 0,0529 | 0,1003 |
| 0,0567 | 0,0667 |
| 0,0425 | 0,0436 |
| 0,0767 | 0,0729 |
| \| **Wilcoxon matched-pairs signed rank test** \|  \| \| --- \| --- \| \| P value \| 0,1094 \| \| Exact or approximate P value? \| Exact \| \| P value summary \| ns \| \| Significantly different (P < 0.05)? \| No \| \| One- or two-tailed P value? \| Two-tailed \| |  |
